# Why only me? A case report of a breast cancer patient with unresolved trauma from a past disaster experience developing a mental disorder

**DOI:** 10.1002/ccr3.6680

**Published:** 2022-12-08

**Authors:** Yudai Kaneda, Arinobu Hori, Yasuhiro Kotera, Masahiro Wada, Toyoaki Sawano, Yoshiaki Kanemoto, Tomohiro Kurokawa, Masaharu Tsubokura, Tetsuya Tanimoto, Tomozo Ejiri, Norio Kanzaki, Akihiko Ozaki

**Affiliations:** ^1^ School of Medicine Hokkaido University Sapporo Japan; ^2^ Hori Mental Clinic Minamisoma Japan; ^3^ Faculty of Medicine and Health Sciences University of Nottingham Nottingham UK; ^4^ Department of Breast and Thyroid Surgery Jyoban Hospital of Tokiwa Foundation Iwaki Japan; ^5^ Department of Breast Surgery Sano Kosei General Hospital Sano Japan; ^6^ Department of Surgery Jyoban Hospital of Tokiwa Foundation Iwaki Japan; ^7^ Department of Radiation Health Management Fukushima Medical University School of Medicine Fukushima Japan; ^8^ Department of Medical Epigenomics Research Fukushima Medical University Fukushima Japan; ^9^ Department of Internal Medicine Jyoban Hospital of Tokiwa Foundation Iwaki Japan; ^10^ Department of Gastrointestinal Surgery Fukushima Medical University School of Medicine Fukushima Japan

**Keywords:** breast cancer, disaster, Japan, mental health, self‐compassion

## Abstract

Little is known about how the psychological stress of having experienced a natural disaster affects cancer patients. We experienced a patient who was treated with breast cancer after having been stricken by a typhoon, which resulted in significant psychological damage. Treatment strategies should incorporate patients' mental health appropriately after disasters.

## Abstract 2

Given that most breast cancer patients are vulnerable to stress, treatment strategies that can reduce the psychological damage, both in the short and long term, such as implementing social engagement programs or self‐compassion training, have the potential to improve their management.

## 1 INTRODUCTION

Breast cancer is the most common cancer in women, with an estimated 685,000 deaths in 2020.^1^ However, in developed countries, the survival rate exceeds 80%, thanks to its treatment and screening program development.^2^

Breast cancer patients often encounter a variety of psychological challenges. One example is mood disturbances, reported in 90% of the patients, with more than 50% moderate to very severe.^3^ Moreover, their mental disturbances may persist for years even after treatment,^4^ because of changes in body image and sexuality, lowered self‐esteem, and anxiety about cancer recurrence or death.^5^

Natural disasters are one of the most noteworthy examples which are also linked to mental disorders among survivors. It has been reported that a variety of personal and external factors are associated with post‐disaster mental disorders,^6^ such as post‐traumatic stress disorders, depression, and anxiety.^7^ In addition, the survivors often face continuous emotional stress from post‐disaster cleanup, loss of property or employment, and financial struggles.^8^ These factors may have an additional impact on the mental health of breast cancer patients. Therefore, it is important to understand the physical and mental health status of breast cancer patients both in the short and long term, and to provide necessary treatment timely, taking into account their past disasters experiences.

However, the psychological impact of successive exposure to the stressful events of natural disasters and breast cancer treatment has not been sufficiently reported. Here, we report a case of a patient whose treatment for breast cancer after a typhoon experience has caused significant psychological damage.

## 2 CASE PRESENTATION

In April 2021, a breast cancer patient in her 40s, who had no history of drinking or smoking and worked part‐time at a bakery, living with her husband and two children, visited our hospital in Iwaki City, Fukushima, Japan, because her tumor had been receiving treatment at another clinic for about two years but had begun to grow. After various examinations, including mammography, ultrasound, contrast‐enhanced MRI, and PET‐CT, she was diagnosed with cT1cN0M0 Stage1 breast cancer. She underwent mastectomy and sentinel lymph node biopsy approximately three weeks after her initial visit.

After the mastectomy, her husband felt that she seemed to be doing well for a while. However, when she saw and heard the news of typhoons in mid‐September 2021, she began to feel vague anxiety. The patient's detailed story revealed a strong traumatic experience in a past natural disaster. Typhoon Hagibis in October 2019 flooded right in front of her home, forcing her to evacuate. Indeed, this typhoon caused 128 sections of 71 rivers nationwide to collapse due to excessive rain, resulting in the deaths of about 100 people, mostly from drowning. According to her husband, she had always been an earnest person, and even during this typhoon two years ago, her family evacuated to a junior high school on higher ground because she thought the water would fill the canals and submerge their car, but there, she felt intense fear. She could not concentrate and began to suffer from a strong feeling of isolation, wondering, “Why only me?”. Also, we carefully obtained the patient's information in the course of the usual consultations and found that she was less interested in the outside environment and more concerned with her own inner experience, even though this period was a time when COVID‐19 infectious diseases were spreading in Japan.

In October 2021, she visited another psychiatric clinic. After the examination, her condition was determined to be depression, with the following symptoms: anxiety, palpitation, emotional disturbance, agitation, fatigue, loss of concentration, sleep disturbance, and appetite loss. Indeed, her CES‐D scored 32. However, since the patient's psychomotor inhibition was mild and her communication and mood reactivity were maintained, she was not considered to have severe depression but rather a strong reactivity to trauma and loss experiences. Therefore, tandospirone, classified as anxiolytics, was selected as the medication rather than a standard antidepressant, and 30 mg of it was prescribed. In addition to medication, psychoeducation with an awareness of post‐traumatic growth was also implemented as a key part of the treatment.^9^ Specifically, she and her husband were informed in detail about the possible causes of her depression: the impact of the lost experience from the mastectomy, the untreated traumatic experience of a typhoon two years ago, the physiological impact of the postoperative hormone therapy, and about the possible influence of her serious nature. They were convinced of these explanations and followed the instruction to take a period of rest so that she could allow herself to be unwell. With the support of her family and outpatient psychiatry visits every two months, this patient recovered her mental stability as of March 2022. Although there was a big earthquake in Iwaki City on March 16, 2022, she expressed that her anxiety then was as much as everyone else's and did not feel any palpitations. In the meantime, the prescription of tandospirone which had been reduced to 20 mg was returned to 30 mg just in case.

## 3 DISCUSSION

We reported a case in which the effects of the past natural disaster might have worsened the psychological stress of breast cancer treatment. Although the number of studies examining the influence of disasters on cancer treatment is increasing, the psychological effects have not gained enough attention as an additional factor. Our case highlights the importance of evaluating psychological effects by the disaster in daily practice, as it has been noted that they may affect the prognosis.^10^

Natural disasters have been reported to cause long‐term trauma.^7^ Also, breast cancer patients face much stress from diagnosis to post‐treatment.^5^ In our case, the combination of these influences may have resulted in an intense form of psychological symptoms. These pre‐existing conditions and mental health conditions are increasingly drawing attention under the COVID‐19 pandemic as having a significant impact on patients' mental health.^11^ Therefore, our patient's insufficiently improved mental status could have been a problem, and at least we had to perform a detailed history taking of previous disaster experiences from the early stage of the treatment.^12^

It is also noteworthy that our patient had a sense of “Why only me?”. This applies to “sense of isolation” in the self‐compassion framework.^13^ Taking a universal humanistic approach to patients, that is, making them aware that they are not the only ones going through such experiences, listening to and sympathizing with patients beyond their medical conditions, and offering life as normal as possible may be effective.^13^ In addition, based on the Trauma‐Informed Care (TIC) concept, understanding the connection between past traumatic experiences and symptoms is important for recovery from psychological disorders.^14^ In our case, the patient was able to continue her everyday life at home, thanks to the family's attentive support in addition to medications. Furthermore, the fact that the patient was convinced by our explanations about trauma from the disaster and the surgical loss experience, thereby cooperating with the treatment, may have significantly impacted the improved prognosis. Considering these two aspects of the patient (i.e., isolation and trauma), self‐compassion intervention was deemed effective in this context. As systematic reviews and meta‐analyses reported that enhancing kindness and understanding toward oneself helps to improve a sense of connection and to accept one's current situation.^15^, ^16^, ^17^ Even though generalizability remains to be evaluated further, clinicians can consider this type of intervention when they encounter patients like this case.^18^

Notably, it is necessary to consider that most breast cancer patients are a vulnerable group to stress (women and middle‐aged).^19^ As part of breast cancer treatment, providing narrative‐based medicine (NBM) or having patients interact with others in the same situation may be practical to resolve past trauma and feelings of isolation.^20^

In addition, the findings indicate that the psychological effects of past natural disasters are potentially an essential factor to be considered in the long‐term follow‐up of cancer patients. In our case, her husband happened to be nimble enough to intervene at an early stage, but such a support system is currently not sufficient. While some reports have suggested that the mental impact is limited in cases where a patient feels as if they are improving to some degree from the disaster,^12^ it is important to note that, as in our case, even if a patient appears to be doing well at a glance, cancer treatment may cause her to recall past experiences of disaster, which may lead to psychological and emotional disorders. As the number of cancer survivors increases, it is important to consider past disaster experiences as a risk factor affecting the prognosis of cancer patients. Thus, it is important to prepare a system that can address the psychological needs of patients under the collaboration of multiple professionals, including medical social workers and clinical psychologists.

In conclusion, we reported a case of a patient suffering severe psychological damage from breast cancer treatment after being hit by a typhoon. While the survival rate of breast cancer patients has increased, this case suggests that past disaster experiences can have a negative psychological impact on the postoperative management of breast cancer patients. It should be noted that such cases may increase in the future, especially in disaster‐prone countries like Japan. Likewise, the increase in natural disasters caused by climate change and other factors could also happen in other countries besides Japan. Rather than focusing solely on cancer treatment with individualized therapies, it is important to gather information about the internal and external factors surrounding the patient through routine consultations and make holistic treatment strategies, including an intervention that can reduce psychological stress, such as offering social engagement programs or implementing self‐compassion training from the early stages of breast cancer treatment.

## AUTHOR CONTRIBUTIONS

**Yudai Kaneda:** Conceptualization; writing – original draft. **Arinobu Hori:** Writing – review and editing. **Yasuhiro Kotera:** Writing – review and editing. **Masahiro Wada:** Writing – review and editing. **Toyoaki Sawano:** Writing – review and editing. **Yoshiaki Kanemoto:** Writing – review and editing. **Tomohiro Kurokawa:** Writing – review and editing. **Masaharu Tsubokura:** Conceptualization; writing – review and editing. **Tetsuya Tanimoto:** Writing – review and editing. **Tomozo Ejiri:** Writing – review and editing. **Norio Kanzaki:** Writing – review and editing. **Akihiko Ozaki:** Supervision; writing – review and editing.

## FUNDING INFORMATION

None.

## CONFLICT OF INTEREST

Akihiko Ozaki receives personal fees from MNES Inc, outside the submitted work. Also, Tetsuya Tanimoto receives personal fees from MNES Inc, and Bionics co., ltd. outside the submitted work. No other authors reported conflicts of interest.

## CONSENT

Written informed consent was obtained from the patient to publish this report in accordance with the journal's patient consent policy.

## Data Availability

None.
